# Diethylstilbestrol, a Novel ANO1 Inhibitor, Exerts an Anticancer Effect on Non-Small Cell Lung Cancer via Inhibition of ANO1

**DOI:** 10.3390/ijms22137100

**Published:** 2021-07-01

**Authors:** Yohan Seo, Sung Baek Jeong, Joo Han Woo, Oh-Bin Kwon, Sion Lee, Hye In Oh, Sungwoo Jo, Seon Ju Park, Wan Namkung, Uk Yeol Moon, Sungwoo Lee

**Affiliations:** 1 New Drug Development Center, Daegu Gyeongbuk Medical Innovation Foundation, Daegu 41061, Korea; sjeong100@dgmif.re.kr (S.B.J.); kob325@dgmif.re.kr (O.-B.K.); sionlee@dgmif.re.kr (S.L.); bluemuy@dgmif.re.kr (U.Y.M.); 2 Department of Physiology, Dongguk University College of Medicine, Gyeongju 38066, Korea; gabriel929@dongguk.ac.kr; 3 Channelopathy Research Center (CRC), Dongguk University College of Medicine, 32 Dongguk-ro, Ilsan Dong-gu, Goyang 10326, Korea; 4 Underwood Division Economics, Underwood International College, Yonsei University, 50 Yonsei-ro, Seodaemun-gu, Seoul 03722, Korea; arielleohhyein@yonsei.ac.kr; 5 College of Pharmacy and Yonsei Institute of Pharmaceutical Sciences, Yonsei University, 85 Songdogwahak-ro, Yeonsu-gu, Incheon 21983, Korea; dsdyu2005@naver.com (S.J.); wnamkung@yonsei.ac.kr (W.N.); 6 Chuncheon Center, Korea Basic Science Institute (KBSI), Chuncheon 24341, Korea; sjp19@kbsi.re.kr; 7 Interdisciplinary Program of Integrated OMICS for Biomedical Science Graduate School, Yonsei University, Seoul 03722, Korea

**Keywords:** diethylstilbestrol, anoctamin 1, inhibitor, lung cancer, apoptosis

## Abstract

Non-small cell lung cancer (NSCLC) is one of the leading causes of cancer-related mortality; thus, therapeutic targets continue to be developed. Anoctamin1 (ANO1), a novel drug target considered for the treatment of NSCLC, is a Ca^2+^-activated chloride channel (CaCC) overexpressed in various carcinomas. It plays an important role in the development of cancer; however, the role of ANO1 in NSCLC is unclear. In this study, diethylstilbestrol (DES) was identified as a selective ANO1 inhibitor using high-throughput screening. We found that DES inhibited yellow fluorescent protein (YFP) fluorescence reduction caused by ANO1 activation but did not inhibit cystic fibrosis transmembrane conductance regulator channel activity or P2Y activation-related cytosolic Ca^2+^ levels. Additionally, electrophysiological analyses showed that DES significantly reduced ANO1 channel activity, but it more potently reduced ANO1 protein levels. DES also inhibited the viability and migration of PC9 cells via the reduction in ANO1, phospho-ERK1/2, and phospho-EGFR levels. Moreover, DES induced apoptosis by increasing caspase-3 activity and PARP-1 cleavage in PC9 cells, but it did not affect the viability of hepatocytes. These results suggest that ANO1 is a crucial target in the treatment of NSCLC, and DES may be developed as a potential anti-NSCLC therapeutic agent.

## 1. Introduction

Lung cancer is categorized into two types, namely, non-small cell lung cancer (NSCLC) and small cell lung cancer. Of them, NSCLC is the most prominent cause of cancer-related mortality and accounts for more than 80% of all documented lung cancer cases [1]. In recent years, several attempts to enhance treatment efficiency using molecular or immunological methods by treating lung cancer through targeted therapy or immunotherapy have been reported [1,2,3]. A few therapies have been developed to target epidermal growth factor receptor tyrosine kinase inhibitors (EGFR-TKIs) and programmed cell death protein-1 (PD-1) as therapeutic targets, whereas others have focused on the suppression of abnormal signaling pathways, including the PI3K/AKT/mTOR, RAS/BRAF/MAPK and JAK/STAT pathways [2,3,4]. However, since the pathological mechanism of NSCLC remains unclear, limitations in currently available therapeutic options, such as resistance to treatment, have been encountered [5]. As a result, there has been an increasing demand for new anticancer targets, as well as the development of novel therapeutic agents.

The Ca^2+^-activated Cl^−^ channel (CaCC) is regarded to be a new anticancer target owing to its role in controlling epithelial cell secretion and cell volume regulation [6,7]. Anoctamin1 (ANO1), a major CaCC, has recently been reported to be overexpressed in gastrointestinal, prostate, head and neck, breast, and colon carcinomas [8,9,10,11]. ANO1 contributes to cancer development via the promotion of oncogenic signaling, including the EGFR and CAMK pathways [8,12]. Particularly, evidence has shown that ANO1 and EGFR form a functional complex that promotes the development of head and neck cancers [13].

Hence, ANO1 may be considered an important target for the treatment of various cancers; however, its role in NSCLC has not been fully elucidated, nor have anticancer drug candidates capable of inhibiting ANO1 been identified. Although pharmacological inhibition of ANO1 reportedly inhibits the growth of various cancer cells [14,15,16,17,18,19], the potency, selectivity, safety, stability and mechanism of action of ANO1 inhibitors in NSCLC have not been identified [14,15,16,17,18,19]. Because of the complexity of the drug development process, discovering lead compounds capable of effectively inhibiting ANO1 is a challenging task.

In this study, diethylstilbestrol (DES), a synthetic estrogen approved by the FDA and a drug used for the treatment of castrate-resistant prostate cancer (CRPC) [20], was newly discovered to be an ANO1 inhibitor. Low-dose DES administration has been reported to be safe and effective in a male population with CRPC, despite them being subjected to anti-androgen therapy [21], indicating the possibility for other drug therapies involving DES. Therefore, this study investigated the potential therapeutic effect and efficacy of DES in NSCLC by screening DES using high-throughput screening and examining the anticancer effect exerted by it on ANO1.

## 2. Results

### 2.1. Identification of a Novel ANO1 Inhibitor Using a High-Throughput Yellow Fluorescent Protein Reduction Assay

**Figure 1:**
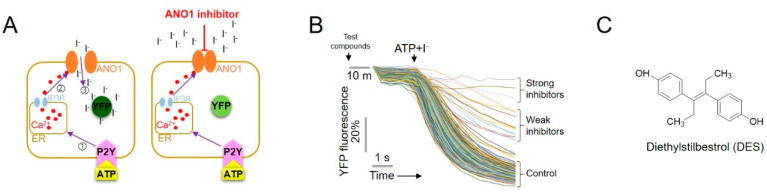
High-throughput YFP reduction assay used to screen for ANO1 inhibitors. (**A**) Cell-based high-throughput YFP reduction assay. ATP-induced P2Y receptor increased calcium levels, which in turn activated ANO1, causing an influx of iodide and a decrease in YFP. (**B**) YFP fluorescence monitored using 96-well plates showing the inhibitory effect of strong inhibitors. (**C**) Chemical structure of the ANO1 inhibitor, diethylstilbestrol (DES).

A cell-based yellow fluorescent protein (YFP) reduction assay was performed to identify a novel ANO1 inhibitor from the FDA-approved drug library. ANO1 chloride channel function was assessed using Fischer rat thyroid (FRT) cells expressing P2Y, ANO1 and YFP (F46L/H148Q/I152L) [22]. P2Y-activation induces an increase in Ca^2+^ ions that activate ANO1 and cause an iodide influx [15,22]. Since the influx of iodide ions quenches YFP fluorescence, the inhibition of ANO1 inhibits the quenching of YFP fluorescence. Thus, the inhibitory effect of the test compounds on ATP-P2Y-Ca^2+^-ANO1 activity was measured using the YFP quenching assay in FRT-ANO1-YFP cells (Figure 1A). Approximately 1300 test compounds were added to the cells for 10 min, after which the cells were treated with ATP and iodide to screen and identify novel ANO1 inhibitors (Figure 1B). A few strong inhibitors inhibited ANO1 activity by >90% at a concentration of 25 μM. One such potent inhibitor was DES, the chemical structure of which is presented in Figure 1C.

### 2.2. Characterization of the Novel ANO1 Inhibitor, DES

**Figure 2:**
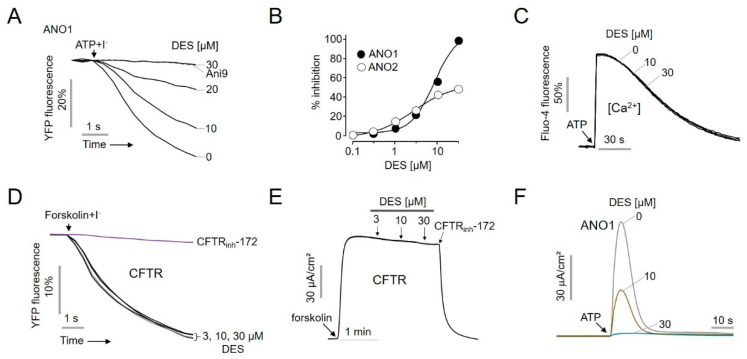
Characterization of the ANO1 inhibitor, diethylstilbestrol (DES). (**A**) Inhibitory effect of DES exerted on ANO1 channel activity determined using YFP fluorescence. (**B**) Summary of dose-response (mean ± S.D., *n* = 5). (**C**) Cytosolic Ca^2+^ level was monitored using Fluo-4 NW in CHO-K1 cells. The indicated concentrations of 10 and 30 μM DES were added for 20 min, and then treatment with 100 μM ATP was performed. (**D**,**E**) Effect of DES on human CFTR activity was measured in FRT-hCFTR-YFP cells using YFP fluorescence and an Ussing chamber. The CFTR was activated in the presence of 10 μM forskolin, whereas its activity was blocked in the presence of 10 μM CFTR_inh_-172. (**F**) Apical membrane currents in FRT-hANO1 cells treated with DES for 10 min prior to ATP-induced ANO1 activation.

To investigate the inhibitory effect of DES on ANO1 chloride channel activity, different doses of DES or 10 μM Ani9 (a known ANO1 channel inhibitor) were administered, and the decrease in YFP fluorescence was measured (Figure 2A). DES inhibited ANO1 channel function in a dose-dependent manner, and dose–response analysis showed that DES completely inhibited ANO1 channel function at an IC_50_ of 6.58 μM, although it only partially inhibited ANO2 channel function (Figure 2B).

To verify whether DES exerted an effect on intracellular Ca^2+^ levels, CHO-K1 cells were loaded with Fluo-4, a fluorescent Ca^2+^ dye. DES was added at concentrations of 10 and 30 μM for 20 min, after which 100 µM ATP was added to increase Ca^2+^ levels via P2Y signaling. The presence of up to 30 µM DES did not affect the ATP-induced intracellular Ca^2+^ increase in CHO-K1 cells that did not express ANO1 (Figure 2C). To elucidate whether DES affected other chloride channels, such as the cystic fibrosis transmembrane conductance regulator (CFTR), YFP fluorescence and short-circuit currents were measured in FRT cells expressing human CFTR. DES was found to have no effect on CFTR channel function at concentrations up to 30 μM (Figure 2D), and forskolin-induced CFTR activity was inhibited by CFTR_inh_172 (a known CFTR channel inhibitor) (Figure 2E).

To examine the direct inhibition of ANO1 channel activity by DES, the inhibition of short-circuit currents by ANO1 activation was verified. Results showed that DES selectively inhibited ANO1-induced current in a dose-dependent manner (Figure 2F), indicating that DES inhibited the secretion of chloride ions via activation of the ANO1 channel.

**Figure 3:**
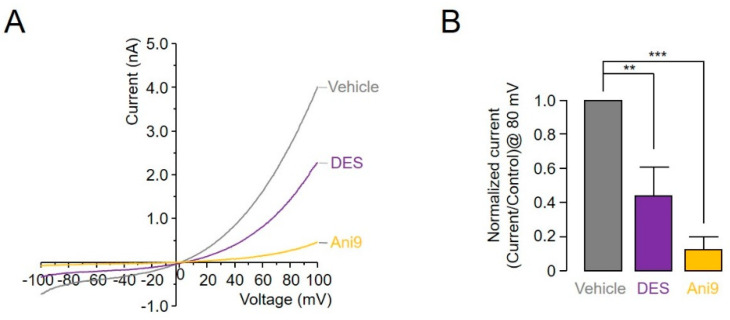
DES and Ani9 inhibit ANO1-induced currents in ANO1-expressing HEK293 cells. (**A**) Current/voltage (I/V) plot of mean currents. (**B**) Bar graph summarizing current densities measured at +80 mV (mean ± S.D., *n* = 5–8). ** *p* < 0.01, *** *p* < 0.001, Students’ unpaired *t*-test.

Additionally, a whole-cell patch clamp was performed to confirm whether DES inhibited ANO1 channel function in single cells. ANO1 currents were recorded in HEK293 cells transiently expressing human ANO1. Results showed that Ani9 and DES significantly suppressed the ANO1-induced current by 77.7% ± 12.5% and 54.8% ± 16.7% at concentrations of 1 and 10 μM, respectively (Figure 3A,B).

### 2.3. ANO1 Protein Levels Decrease in the Presence of DES

**Figure 4:**
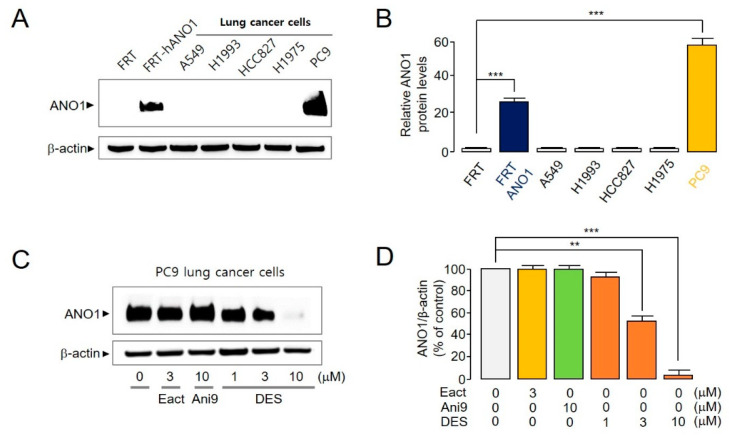
ANO1 expression levels in lung cancer cell lines, and the effect of an ANO1 activator (Eact) and inhibitors (Ani9 and DES) on the PC9 lung cancer cell line. (**A**) Western blot results of ANO1 protein expressed in FRT, FRT-hANO1, A549, H1993, HCC827, H1975 and PC9 cells. (**B**) Relative ANO1 protein levels (mean ± S.D., *n* = 5). (**C**) PC9 cells were subjected to treatment with Eact, Ani9, or DES; ANO1 protein levels were measured after 72 h. (**D**) ANO1 protein intensities were normalized to β-actin (mean ± S.D., *n* = 5). ** *p* < 0.01, *** *p* < 0.001.

ANO1 overexpression and function have been reported in a variety of carcinoma cells [14,15,16,17,18,19]; however, the expression and function of ANO1 in lung cancer cells has not been comprehensively studied [23]. To measure the expression level of ANO1 in various lung cancer cell lines, we confirmed endogenous ANO1 protein expression levels in FRT cells, FRT cells stably expressing ANO1, and various lung cancer cell lines (Figure 4A). Notably, the ANO1 expression level in PC9 lung cancer cells was twice as high as that in the protein sample derived from FRT cells stably overexpressing ANO1 (Figure 4B). Thus, PC9 cells expressing the highest level of ANO1 were subjected to treatment with Eact (ANO1 channel activator), Ani9 and DES for 72 h, and changes in ANO1 protein expression levels were measured (Figure 4C). Eact and Ani9 did not alter ANO1 protein expression levels, whereas concentrations of 3 and 10 μM DES reduced ANO1 expression by approximately 48.5% and 97%, respectively (Figure 4D). Therefore, we confirmed that DES not only inhibited the function of the ANO1 channel, but it also potently reduced the expression level of ANO1.

### 2.4. Inhibitory Effects of DES on Cell Proliferation and Migration in PC9 Lung Cancer Cells

**Figure 5:**
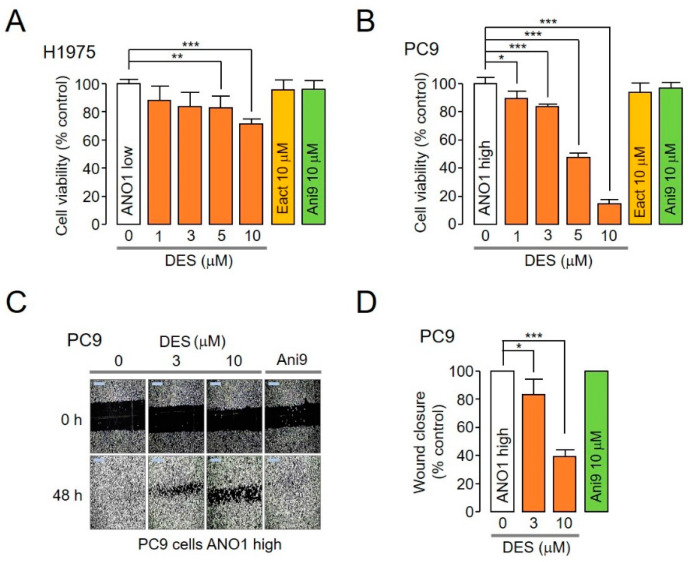
Effect of DES, Eact and Ani9 on cell viability and migration. (**A**,**B**) H1975 cells expressing low levels of ANO1 and PC9 cells expressing high levels of ANO1 were subjected to treatment with DES, Eact or Ani9 at the indicated concentrations; cell viability was determined after 72 h using the CCK-8 assay (mean ± S.D., *n* = 5). (**C**,**D**) Wound-healing assay was performed using PC9 cells expressing high levels of ANO1. The cells were subjected to treatment with 3 μM DES, 10 μM DES, and 10 μM Ani9, and representative images were acquired at 0 and 48 h post wound infliction. (right) The wound closure was quantified at 48 h (mean ± S.D., *n* = 3). * *p* < 0.05, ** *p* < 0.01, *** *p* < 0.001.

Previous studies have reported that pharmacological inhibition of ANO1 inhibits the development of several carcinomas [24]. To confirm whether this effect of the pharmacological inhibition of ANO1 was also applicable to lung cancer cells, we selected H1975 cells that did not express ANO1 and PC9 cells highly expressing ANO1. As indicated in Figure 5, DES, which reduces both ANO1 channel function and protein levels, and Eact (ANO1 activator) and Ani9 (ANO1 inhibitor), which control only ANO1 channel function, were added and subsequently incubated for 72 h, after which cell viability was analyzed. Notably, at concentrations of 3, 5 and 10 μM, DES decreased cell viability by 16.1%, 17.0% and 28.5%, respectively, in H1975 cells and by 15.8%, 51.8% and 85.2%, respectively, in PC9 cells. In contrast, Eact and Ani9 did not inhibit cell viability (Figure 5A,B). Hence, these results showed that DES decreased the viability of PC9 cells by reducing ANO1 protein levels.

To further determine whether DES inhibited the migration of PC9 cells, a wound-healing assay was performed. DES significantly inhibited PC9 cell migration in a dose-dependent manner (Figure 5C,D). Collectively, these results indicate that DES inhibited both cell viability and the migration of NSCLC PC9 cells.

### 2.5. Reduction in ANO1 Protein, p-EGFR and p-ERK1/2 Levels by DES

**Figure 6:**
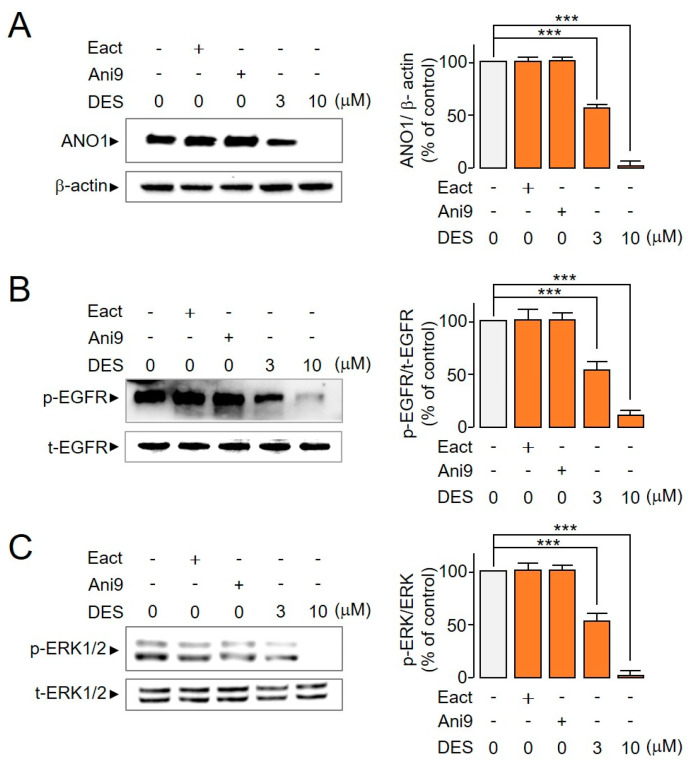
Effect of ANO1 activator (Eact) and inhibitors (Ani9, DES) on ANO1, *p*-EGFR and *p*-ERK1/2 protein levels. (**A**) Immunoblot-mediated detection of ANO1 in PC9 cells expressing high levels of ANO1. The cells were subjected to treatment with the indicated concentrations of Eact, Ani9, or DES for 72 h. (right) ANO1 protein intensities were normalized to those of β-actin (mean ± S.D., *n* = 5). (**B**) The levels of phosphorylation of EGFR in the presence of ANO1 modulators. (right) The *p*-EGFR band intensities were normalized to those of t-EGFR (mean ± S.D., *n* = 5). (**C**) The levels of phosphorylation of ERK1/2 in the presence of ANO1 modulators. (right) The *p*-ERK1/2 levels were normalized to those of t-ERK1/2 (mean ± S.D., *n* = 5). *** *p* < 0.001.

Several ANO1-related signaling pathways, including EGFR-mediated AKT/SRC/ERK1/2 and Ras-Raf-MEK-ERK1/2, have been reported in cancer development [12,13,25]. In NSCLC cells, we investigated the signaling mechanism underlying the decrease in ANO1 protein levels with DES treatment. As ANO1 reportedly establishes direct interactions with EGFR to phosphorylate EGFR and ERK1/2 [8,13], we confirmed that DES significantly decreased ANO1 protein levels (Figure 6A), followed by phosphorylation of EGFR (Figure 6B) and ERK1/2 (Figure 6C). However, Eact and Ani9, which activate or inhibit the function of ANO1 channels, neither decreased the protein levels of ANO1 nor inhibited the activation of ERK1/2 and EGFR (Figure 6). These results showed that DES decreased ANO1 protein levels, leading to the inhibition of EGFR activation and the subsequent inhibition of ERK1/2 activation.

### 2.6. Apoptotic Effect and Hepatocytotoxicity of DES

**Figure 7:**
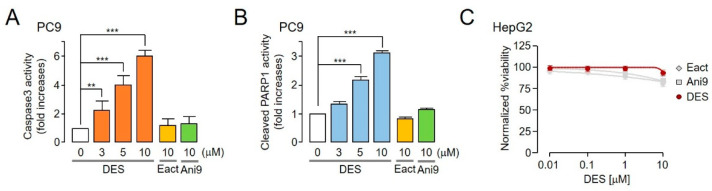
Apoptotic effect and hepatocytotoxicity of DES. (**A**) PC9 cells cultured with DES, Eact and Ani9 at the indicated concentrations for 24 h, with subsequent assessment of caspase-3 activity (mean ± S.D., *n* = 5). (**B**) The cells were cultured with the indicated concentrations of compounds for 24 h, and then cleaved PARP-1 activity was measured (mean ± S.D., *n* = 5). (**C**) DES concentration-dependent relative viability of HepG2 cells (red) was measured by quantifying resorufin derived from resazurin by intracellular reductases. Control experiments (Eact and Ani9) are marked for comparative analysis (gray) (mean ± S.D., *n* = 8). ** *p* < 0.01, *** *p* < 0.001.

Pharmacological inhibition of ANO1 reportedly induces apoptosis in various cancer cells [26]. Thus, we verified the extent to which DES induced apoptosis in PC9 lung cancer cells. DES significantly increased caspase-3 activity and PARP-1 cleavage, a hallmark of apoptosis (Figure 7A,B). Meanwhile, Eact and Ani9 exerted no effect on caspase-3 activity and PARP-1 cleavage, as expected. These results revealed that DES exhibited anticancer effects via apoptosis as well as ANO1 reduction. Furthermore, the indicated concentrations of Eact, Ani9 and DES were added to hepatocytes (HepG2) to verify the potential applicability of DES as a drug for NSCLC. The results showed that DES did not inhibit HepG2 cell viability at concentrations up to 10 μM (Figure 7C).

## 3. Discussion

The typical physiological roles of ANO1 include the activation of the ANO1 channel and the efflux of Cl^−^ ions. It has been demonstrated to be involved in the treatment of cystic fibrosis, dry mouth syndrome, and dry eye diseases [6,15,24]. However, ANO1 overexpression is a critical target in various carcinomas [8,10,12]. It is highly expressed in prostate carcinoma, glioblastoma, gastrointestinal stromal tumor, esophageal squamous cell carcinoma (ESCC), and head and neck squamous cell carcinoma (HNSCC), thus activating oncogenic signaling and promoting the growth and migration of cancer cells [8,10,12,27,28,29]. RNAi-related ANO1 knockdown has also been reported to inhibit cell growth and cause apoptosis in several xenograft studies [8,10,12,27,28,29]. Therefore, a reduction in ANO1 protein expression can result in various adverse effects, such as dry mouth, dry eye, low blood pressure (hypotension) and intestinal dysmotility [6,15,24,30,31,32]; nonetheless, this reduction presents with a considerable advantage in terms of anticancer effects.

ANO1 was first cloned in 2008 [33]; nevertheless, the development ANO1 inhibitors as drugs is seemingly advancing at a reduced pace owing to the low availability of anion channel studies and the advanced experimental skills required to conduct relevant studies. Ani9, an ANO1 inhibitor, has been found to potently inhibit the function of the ANO1 channel [15]. However, in our study, it did not reduce ANO1 protein levels (Figure 4). This weak effect of Ani9 on cell viability was attributed to its low plasma stability [15,18]. As the structure–activity relationship was considered to improve the plasma stability of Ani9, 5f was newly synthesized [15,18]. The plasma stability of 5f was two-fold higher than that of Ani9, and 5f decreased the ANO1 protein level by approximately two-fold more than Ani9. Thus, the viability of cancer cells was more strongly reduced by treatment with 5f than with Ani9 [15,18]. Nonetheless, 5f had almost no effect on the tumor in a xenograft mouse model (data not shown). Despite being a difficult and time-consuming process, the improvement of physical properties of a compound is necessary because a substantial number of patients worldwide rely upon, and a considerable market exists for, the development of an effective treatment for lung cancer [34].

Therefore, to develop a lead compound for lung cancer treatment, we used an FDA-approved drug library with excellent properties and safety measures in places and discovered a hit compound, DES, that inhibits ANO1 and which is currently used as a prostate cancer treatment agent. Measurement of the viability of PC9 cells after DES and Ani9 treatments showed that Ani9 had lower stability and less remarkable effects on the inhibition of cell viability than all tested concentrations of DES. As expected, the results of this study showed that DES strongly reduced the viability of PC9 cells by decreasing ANO1 protein levels. Nevertheless, further study is needed to elucidate the mechanism by which DES decreases the amount of ANO1 protein, as well as the site at which DES binds to ANO1.

The high expression of ANO1 in HNSCC, ESCC, and prostate cancer reportedly increases metastasis and decreases the survival rate of patients [9,27,35]. However, it is not clear whether ANO1 contributes to cancer development by releasing chloride through ANO1 activation or whether ANO1 is a signal transduction of cancer development through protein expression of ANO1 [15,19,35]. To address this issue, in this study, we investigated whether the activation of the ANO1 channel increases cell viability. Our results showed that Eact did not increase ANO1 protein levels and exerted no effect on cell viability (Figure 6A). This supports the hypothesis that DES inhibits the growth and migration of NSCLC PC9 cells by reducing ANO1 protein expression. In addition, several mechanisms of cancer development are related to ANO1, such as the EGFR-mediated AKT/SRC/ERK1/2 or Ras-Raf-MEK-ERK1/2 pathways; nonetheless, the underlying mechanism is unclear in NSCLC [8,13]. We confirmed that DES reduced ANO1 protein expression and inhibited the growth of PC9 cells by inhibiting EGFR activation and ERK1/2 activation (Figure 6). Moreover, DES inhibited PC9 cell migration, but Eact and Ani9 did not affect cell viability or the phosphorylation of EGFR and ERK1/2. Notably, even though the epithelial growth factor was not added, endogenous ANO1 protein expression seemed to increase the phosphorylation levels of EGFR (Figure 6). Therefore, the reduction in ANO1 protein, *p*-ERGR and *p*-ERK1/2 levels by DES is important for the inhibition of cell viability and the migration of PC9 cells expressing high levels of ANO1. Furthermore, DES significantly induced apoptosis via an increase in caspase-3 activity and PARP-1 cleavage, a hallmark of apoptosis (Figure 7).

As described above, DES inhibited the phosphorylation of EGFR by reducing ANO1 protein levels and inhibited the phosphorylation of ERK1/2. However, Eact and Ani9, which regulate channel functions, exerted no effect on apoptosis because they did not reduce ANO1 protein expression. Therefore, it seems that DES demonstrates anticancer effects through the reduction in ANO1 protein expression, EGFR and ERK1/2 phosphorylation, and apoptosis. As DES is an FDA-approved drug, additional study is necessary to assess the effect of DES alone and in combination with drugs used in NSCLC treatment. If DES is to be co-administered with other drugs, further study should be performed to examine any safety concerns and whether there is a change in its inhibitory effect in NSCLC.

## 4. Materials and Methods

### 4.1. Material and Solutions

FDA-approved drugs were provided by Dr. Sang-Hyun Min at the Daegu Gyeongbuk Medical Innovation Foundation (DGMIF, Daegu, Korea). Eact and Ani9 were purchased from Tocris Bioscience (Bristol, UK). DES was purchased from Sigma-Aldrich (St. Louis, MO, USA).

### 4.2. Cell Culture

FRT cells stably expressing ANO1 and CFTR were provided by Alan Verkman (University of California, San Francisco, CA, USA) and cultured in Coon’s modified F12 medium with 10% fetal bovine serum (FBS), 2 mM L-glutamine, 100 units/mL penicillin, and 100 μg/mL streptomycin. HEK293T, A549, H1993, HCC827, H1975 and PC9 cells were provided by the New Drug Development Center (DGMIF, Korea). HEK293T cells were cultured in the Dulbecco’s modified Eagle medium (DMEM), while other cells were cultured in RPMI-1640 medium supplemented with 10% FBS, 100 units/mL penicillin, and 100 μg/mL streptomycin.

### 4.3. High-Throughput Screening Using a YFP Reduction Assay

YFP variant (YFP-H148Q/I152L/F46L)- and ANO1-expressing FRT cells were cultured in 96-well microplates at a confluence of ~90% per well. After 24 h, wells were subjected to washing with PBS and treated with the test compounds for 10 min. The YFP in each well was monitored every 0.5 s for 8 s by using the Synergy neo microplate reader. To monitor iodide influx via the ANO1 channel, 100 μL of 70 mM iodide solution with 100 μM ATP was added by using an injector in a microplate reader (Synergy neo2, BioTek, Winooski, VT, USA) to all 96 wells, 2 s after the initiation of YFP fluorescence measurements. The inhibitory effect of test compounds exerted on ANO1 channel activity was measured using the initial slope generated for the decrease in YFP.

### 4.4. Cytosolic Ca^2+^ Measurement

CHO-K1 cells were cultured in 96-well black-walled microplates and loaded with Fluo4 NW according to the manufacturer’s instructions (Invitrogen, Carlsbad, CA, USA). Briefly, CHO-K1 cells were incubated with 100 μL of Fluo4 NW mixture (1X Hanks’ balanced salt solution with 2.5 mM probenecid and 20 mM HEPES) including Fluo-4. After 40 min of incubation in the dark, the 96-well plates were transferred to a plate reader to conduct the fluorescence assay. Fluo-4 fluorescence was measured using the Synergy neo2 microplate reader (BioTek) equipped with syringe pumps and custom Fluo-4 excitation/emission filters (485/538 nm).

### 4.5. Ussing Chamber-Based Analysis

FRT cells expressing ANO1 and CFTR were mounted in Ussing chambers (Physiologic Instruments, San Diego, CA, USA). The basolateral bath was filled with HCO_3_^−^ buffered solution containing 120 mM NaCl, 5 mM KCl, 1 mM MgCl_2_, 1 mM CaCl_2_, 10 mM D-glucose, 2.5 mM HEPES and 25 mM NaHCO_3_ (pH 7.4), and the apical bath was filled with a half-Cl^−^ solution. In the half-Cl^−^ solution, 65 mM NaCl in the HCO_3_^−^ buffered solution was replaced with Na-gluconate. The basolateral membrane was then permeabilized with 250 μg/mL amphotericin B. Cells were incubated in bath solutions for 20 min and aerated with 95% O_2_/5% CO_2_ at 37 °C. Forskolin was applied to the apical membrane to activate CFTR, ATP was applied to the apical membrane to activate ANO1 via P2Y receptor-induced Ca^2+^ increase, and test compounds were applied to both apical and basolateral bath solutions 20 min before ANO1 and CFTR activation. Apical membrane currents were measured using the EVC4000Multi-Channel V/I Clamp (World Precision Instruments, Sarasota, FL, USA) and a Power Lab 4/35 (AD Instruments, Castle Hill, Australia). Data were analyzed using Lab Chart Pro 7 (AD Instruments). The sampling rate was set at 4 Hz.

### 4.6. Whole-Cell Patch Clamp Experiments

Patch clamp recordings were performed for HEK-293 cells transiently expressing ANO1. The pipette solution contained 150 mM N-methyl-D-glucamine-Cl (NMDG-Cl), 1 mM MgCl_2_, 3 mM MgATP, 5 mM HEPES, 10 mM ethylene glycol tetraacetic acid and 6.6 mM CaCl_2_ (pH 7.2) (300 nM Ca^2+^). The bath solution was composed of 150 mM NMGD-Cl, 1 mM CaCl_2_, 1 mM MgCl_2_, 5 mM glucose and 10 mM HEPES (pH 7.4). The pipettes were prepared using borosilicate glass and had resistances of 3–5 MΩ after subjection to fire polishing. Seal resistances were between 3 and 10 GΩ. The pulse protocol was as follows: holding potential, 0 mV; ramp pulse, −100 to 100 mV; pulse duration, 1 s; and pulse to pulse interval, 20 s. Recordings were performed at room temperature (RT) using an Axopatch 200 B (Axon Instruments, San Jose, CA, USA). Currents were digitized using the Digidata 1440 A converter (Axon Instruments), and data were filtered at 5 kHz and sampled at 1 kHz.

### 4.7. Western Blot Analysis

Sample preparation was conducted as per methods described previously [36]. Protein samples were separated using 4–20% Mini-PROTEIN TGX^TM^ Precast Gels and transferred onto nitrocellulose membranes (0.45 μm) using the Trans-Blot Turbo Blotting System (Bio-Rad, Hercules, CA, USA). Blocking was performed using the Everyblot Blocking buffer (Bio-Rad). Membranes were then incubated with primary antibodies, containing recombinant anti-TMEM16A antibody [SP31] (ab64085) (Abcam, Cambridge, UK), phospho-p44/42 MAPK (Erk1/2) (Thr202/Tyr204) antibody #9101 (cell signaling, Danvers, MA, USA), p44/42 MAPK (Erk1/2) antibody #9102 (cell signaling), phospho-EGF Receptor (Tyr1068) (D7A5) XP^®^ rabbit mAb #3777 (cell signaling), anti-EGFR antibody (A-10) (Santa Cruz Biotechnology, Dallas, TX, USA), and anti-β-actin (Santa Cruz Biotechnology) overnight. The membranes were then incubated with HRP-conjugated anti-secondary IgG antibodies (Enzo Life Science, Farmingdale, NY, USA) for 1 h at RT. Finally, visualization was performed using the Clarity^TM^ Western ECL substrate with ChemiDoc (Bio-Rad).

### 4.8. Cell Viability Assessment

Cell viability assays were performed using the Cell Counting Kit-8 (Dojindo, Rockville, MD, USA). PC9 and H1975 cells (2 × 10^3^ cells/well) were seeded in 96-well plates and re-cultured with medium (2.5% FBS) containing various concentrations of compounds over approximately 3-fold serial dilution series every 24 h for 3 days. After 72 h, CCK-8 solution was added, and the cells were further incubated for 2 h. Absorbance was measured using a microplate reader at 450 nm (Synergy^TM^ Neo, BioTek).

### 4.9. Wound Healing Assay

PC9 cells were seeded at a density of 2 × 10^4^ cells/well and cultured overnight to achieve 90–100% confluence in 96-well Image Lock tissue culture plates (Essen BioScience, MI, USA). Wounds were inflicted in monolayers according to the manufacturer’s instructions. The cells were subjected to washing steps twice with PBS to remove detached cells and then cultured with medium (2.5% FBS) containing the indicated concentrations of compound every 24 h for 2 days. Images were acquired using a bright-field microscope. Data were analyzed by assessing wound confluence, and wound healing was calculated using the ImageJ software (US National Institutes of Health, MD, USA)

### 4.10. Caspase-3/CPP32 Colorimetric Assay

Caspase-3 activity was measured according to the manufacturer’s instructions (#K106, Biovison, Milpitas, CA, USA). PC9 cells were cultured in 6-well plates until they reached 80% confluence. In the corresponding wells, DES, Eact, and Ani9 were added. After incubation for a duration of 24 h, 1 × 10^6^–5 × 10^6^ cells were subjected to lysis using the cell lysis buffer for 10 min on ice. Supernatants were collected after the cells were centrifuged for 5 min at 4 °C. One hundred micrograms of protein/50 μL buffer was added to each well with 2× reaction buffer containing 10 mM DTT. To measure caspase3 activity, 5 μL DEVD-pNA substrate was injected and incubated for 1 h at 37 °C. The optical density was measured at 400 nm using a microplate reader (Synergy^TM^ Neo, BioTek).

### 4.11. Human Cleaved PARP-1 Activity Assay

Cleaved PARP-1 activity was measured according to the manufacturer’s instructions (#ab174441, Abcam, UK). PC9 cells were cultured in 6-well plates until they reached 70% confluence. DES, Eact, and Ani9 were added to each well. After 24 h, 1 × 10^7^–5 × 10^7^ cells were lysed using the cell extraction buffer for 20 min on ice. Supernatants were collected after the cells were centrifuged (13,000 rpm) for 20 min at 4 °C. Next, 100 μg protein/50 μL buffer was added to each well with an antibody cocktail containing a capture antibody and detector antibody and incubated for 1 h. Wells were washed with 1x wash buffer and then TMB development solution was added with incubation for 10 min. Finally, the stop solution was added, and optical density was measured at 450 nm using a microplate reader (Synergy^TM^ Neo, BioTek).

### 4.12. Hepatocytotoxicity Measurement

HepG2 cells were seeded on a black and clear-bottom 96-well plate in DMEM high glucose medium with 10% (*v*/*v*) FBS, 100 μg/mL penicillin, and streptomycin and cultured at 37 °C with 5% CO_2_ until achieving approximately 70–80% confluency. Test compounds were diluted using the assay medium to concentrations of 0.01, 0.1, 1, 10 and 100 μM with 0.2% (*v*/*v*) DMSO, and 0.2% (*v*/*v*) DMSO solvent and 0.01% (*v*/*v*) Triton X-100 were considered as “no cell death” and “total cell death” control experiments, respectively; incubation for 24 h was conducted. Subsequently, 20% (*v*/*v*) resazurin reagent (#G8080, Promega, Madison, WI, USA) was added to the compound-treated cells and incubated for 2–3 h. The relative amount of resorufin was quantified by measuring the fluorescence intensity at 590 nm. Finally, data were normalized to the control experiments and converted into “Normalized %viability” according to the following equation:

$$\mathrm{Normalized}\;\%\;\mathrm{viability}=\frac{\left(\mathrm{experimental}\;\mathrm{data}-\mathrm{total}\;\mathrm{cell}\;\mathrm{death}\;\mathrm{control}\right)}{\left(\mathrm{no}\;\mathrm{cell}\;\mathrm{death}\;\mathrm{control}-\mathrm{total}\;\mathrm{cell}\;\mathrm{death}\;\mathrm{control}\right)}\times 100$$

### 4.13. Statistical Analysis

All experiments were conducted independently for a minimum of three times. The results for multiple trials are presented as mean ± standard deviation (S.D.). Statistical analysis was performed using the Student’s *t*-test or analysis of variance, as appropriate. Statistical significance was set at *p* < 0.05. GraphPad Prism Software was used to plot the dose–response curve and to calculate IC_50_ values.

## 5. Conclusions

In this study, a novel ANO1 inhibitor, DES, selectively inhibited ANO1 through the absence of changes in CFTR activity and intracellular Ca^2+^ levels. DES significantly decreased the viability and migration of PC9 cells by reducing the expression of phospho-EGFR and phospho-ERK1/2 by reduction in ANO1. In summary, DES induces apoptosis and exerts anticancer effects, suggesting DES is a promising drug candidate for the development of NSCLC therapeutic options.

## Data Availability

The data that support the findings of this study are available from the corresponding author upon reasonable request. Some data may not be made available because of privacy or ethical restrictions.

## Abbreviations

ANO1	Anoctamin1
CaCC	Ca^2+^-activated Cl^−^ channel
CFTR	Cystic fibrosis transmembrane conductance regulator
CRPC	Castrate-resistant prostate cancer
EGFR	Epidermal growth factor receptor
ERK1/2	Extracellular signal-regulated kinases
ESCC	Esophageal squamous cell carcinomas
FRT	Fischer rat thyroid
GIST	Gastrointestinal stromal tumor
HNSCC	Head and neck squamous cell carcinoma
NSCLC	Non-small cell lung cancer
PARP	Poly ADP-ribose polymerase
YFP	Yellow fluorescent protein
